# Guide for phenotype-specific profiling of DNA G-quadruplex-regulated genes

**DOI:** 10.1016/j.xpro.2023.102820

**Published:** 2024-01-09

**Authors:** Zhuoyang Zhao, Jianru Wang, Huichuan Yu, Xiaolin Wang

**Affiliations:** 1 Department of General Surgery (Colorectal Surgery), The Sixth Affiliated Hospital, Sun Yat-sen University, Guangzhou, Guangdong 510655, China; 2 Guangdong Provincial Key Laboratory of Colorectal and Pelvic Floor Diseases, The Sixth Affiliated Hospital, Sun Yat-sen University, Guangzhou, Guangdong 510655, China; 3 Guangdong Institute of Gastroenterology, Guangzhou, Guangdong 510655, China; 4 Biomedical Innovation Center, The Sixth Affiliated Hospital, Sun Yat-sen University, Guangzhou, Guangdong 510655, China; 5 Key Laboratory of Human Microbiome and Chronic Diseases (Sun Yat-sen University), Ministry of Education, Guangzhou, Guangdong 510655, China; 6 Department of Spine Surgery, The First Affiliated Hospital of Sun Yat-sen University, Guangzhou 510080, China; 7 Guangdong Province Key Laboratory of Orthopedics and Traumatology, Guangzhou 510080, China

**Keywords:** Sequence analysis, Genomics, Molecular Biology

## Abstract

DNA G-quadruplex (G4) is a non-canonical four-stranded secondary structure that has been shown to play a role in epigenetic modulation of gene expression. Here, we present a primer on phenotype-specific profiling of DNA G-quadruplex-regulated genes. We provide guidance on *in silico* exploration of G4-related genes and phenotypes, and *in vitro* and *in vivo* validation of the relationship between G4 and phenotype. We describe commonly utilized techniques and detail critical steps involved in determining the phenotype-specific G4-regulated genes for subsequent investigations.

## Graphical abstract

**Figure:**
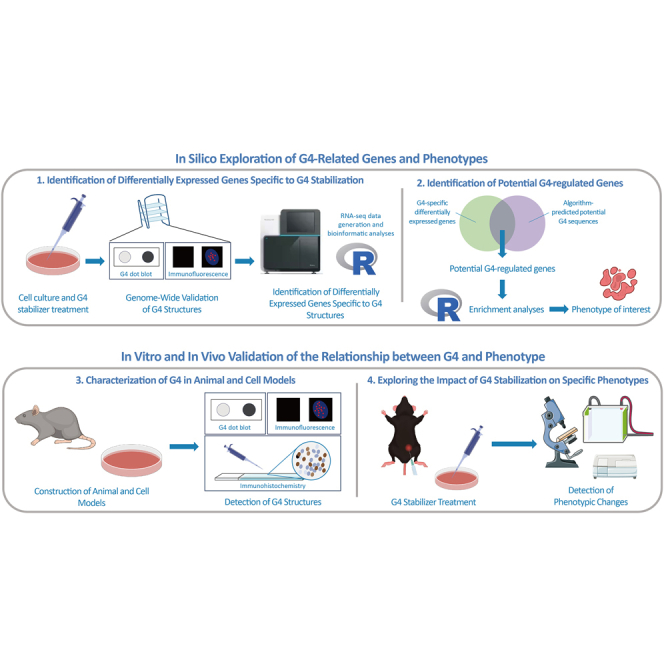

## Background

G-quadruplex (G4) structures are commonly identified within DNA regulatory elements of the mammalian genome and have been recognized for their substantial role in gene expression regulation. The epigenetic influence of G4 structures renders them essential contributors to the determination of cell fate, phenotypic outcomes, and even the onset and progression of diseases. To facilitate the investigation in G4 biology, we present a comprehensive workflow that includes *in silico* inferences, *in vitro* assays, and *in vivo* experiments. By following this workflow, researchers can discern phenotype-specific genes regulated by DNA G4 structures and delve into the effects of G4 on the phenotypes. In this context, we employ the inflammation phenotype in rat intervertebral disc nucleus pulposus (NP) cells as an illustrative example to elucidate this workflow. We employ sequencing data derived from rat NP cells subjected to treatment with or without G4 stabilizers and algorithmically predicted potential G4 sequences (PQS) to define a collection of potential G4-regulated genes. To facilitate *in vitro* and *in vivo* characterization of G4 and its relation to inflammation, we utilize rat NP cells and intervertebral tissue collected from rat models. While this workflow is tailored to rat NP cells, it is reasonable to anticipate the adaptation of this framework for application to other cell lines or organs of interest.

### Institutional permissions

All procedures conducted in this primer were carried out in strict adherence to the ethical guidelines established by Sun Yat-sen University and received thorough review and approval from the Institutional Review Board of Sun Yat-sen University. Additionally, the animal experiments were granted approval by the Animal Experimentation Committee of the Medical Ethics Committee of The Sixth Affiliated Hospital of Sun Yat-sen University (IACUC-2020120102).

## Technical details of the workflow and common techniques

### Compiled dataset of PQS

The algorithmically predicted PQS-containing gene set could be obtained from the Greglist database (http://tubic.tju.edu.cn/greglist). This database offers comprehensive information regarding the quantity, location, and sequence of promoter G4 structures found in genes from various species, including human, rat, mouse, rabbit, zebrafish, and other organisms.

#### Experimental considerations

In light of the inherent limitations associated with algorithmic predictions, we strongly encourage researchers to employ advanced methodologies such as G4-seq or G4 chromatin immunoprecipitation sequencing (G4 ChIP-seq). These techniques should be conducted following well-established protocols to generate a thorough genome-wide mapping of endogenous G4 sequences (EQS).

### Identification of G4-specific differentially expressed genes (DEGs)

This procedure is designed to elucidate alterations in gene expression within NP cells subsequent to the stabilization of genomic G4 structures using G4 stabilizers. It serves as a foundational step for subsequent investigations into gene regulation mediated by G4 structures.

#### Considerations for conducting experiments

Three popular G4 stabilizers could be employed: Pyridostatin (PDS), Phen-DC3, and Braco19. Pyridostatin (PDS) is a widely recognized G4 inducer and stabilizer that interferes with the unwinding of G4-forming DNA by DNA and RNA polymerases, ultimately leading to double-strand breaks. It has been employed to impede the proliferation of human cancer cells. Phen-DC3 is a ligand specifically designed to target G4 structures, exhibiting inhibitory effects on helicases FANCJ and DinG. Braco19 is a telomerase inhibitor renowned for its G4-stabilizing properties, which selectively targets telomeric G4 structures, resulting in DNA damage and cell cycle arrest.

#### Considerations for conducting experiments

**Figure 1:**
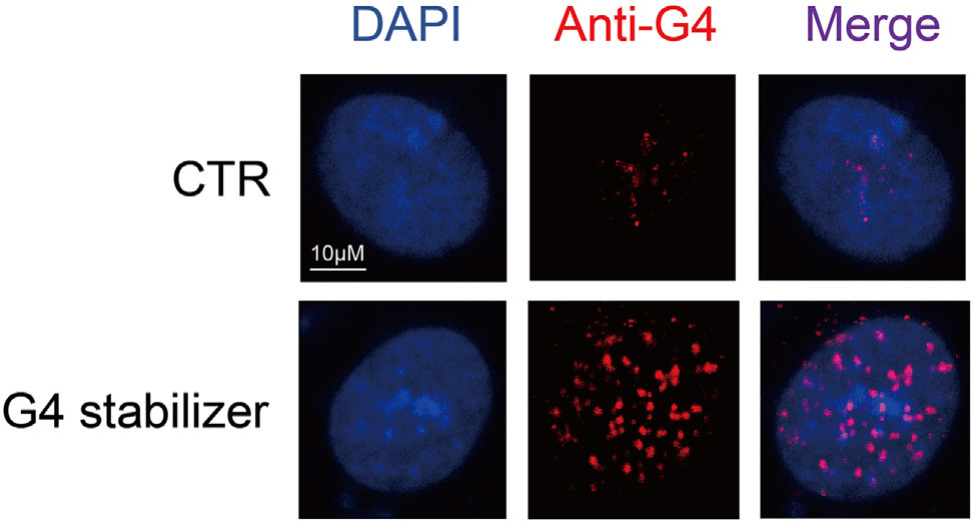
Representative immunofluorescence images for G4 visualization

**Figure 2:**
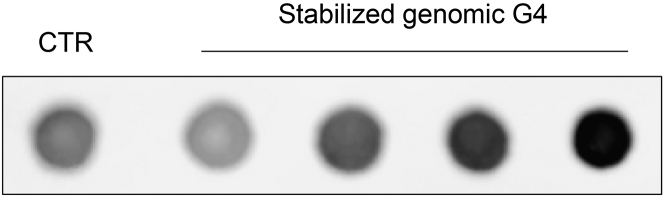
Representative images of dot blot assay for quantification of genomic G4

To evaluate the global G4 stabilization induced by the G4 stabilizers, we recommend the use of immunofluorescence and dot blot techniques. Optional antibodies, including 1H6 and BG4, may be chosen by researchers, both of which have been extensively used for visualizing G4 structures in cells. Figures 1 and 2 provide visual representations of the characteristic changes in genome-wide G4 folding following treatment with G4 stabilizers, as observed through immunofluorescence and dot blot assays.

Following the G4 stabilizer treatment, subsequent steps involve applying sequencing techniques and differential analysis to identify genes that exhibit differential expression. The technical steps involved in this process include RNA extraction, library construction for transcriptome sequencing, and subsequent sequencing and data analysis for differentially expressed genes. See our previously published study for a practical case.

The sequencing data analysis can be performed using the “DESeq2” R package. The genes that demonstrate a significant false discovery rate (FDR) and dramatic fold change are identified as differentially expressed genes (DEGs). The thresholds for significant FDR and dramatic fold change could be optimized to screen the appropriate G4-stabilizer-induced DEGs.

### Identification of potential G4-regulated genes (PQRGs)

Following the introduction of G4 stabilizers, it is important to recognize that the observed changes in gene expression may not exclusively derive from the formation of G4 structures. Hence, the utilization of algorithm inference for PQS is indispensable to minimize the confounding factors that may contribute to the alterations in gene expression. Consequently, the potential G4-regulated genes (PQRGs), which are the intersections of G4-stabilizer-induced DEGs, algorithm-predicted PQS, and EQS if it is available, are generated. See our previously published study for a practical case.

Once the PQRGs have been obtained, the downstream enrichment analysis on this gene set may yield valuable biological insights into the role and mechanism of G4 in phenotype and disease regulation. The enrichment analysis can be effectively executed utilizing the “clusterProfiler” R package; for example, through Gene Ontology (GO) and Kyoto Encyclopedia of Genes and Genomes (KEGG) analyses.

### PQS analysis in promoters of phenotype-specific genes

Following the identification of relevant phenotypes through enrichment analysis, the next critical step is to pinpoint the key genes that establish a link between G4 folding and the observed phenotype changes. These genes will serve as focal points for further investigations.

#### Phenotype-specific gene retrieval

The phenotype-specific gene set could be collected from the prior studies and online databases. In addition, it could be generated from the transcriptomic sequencing techniques and differential analysis following the phenotype-of-interest induction in cell models. As an example, the genes associated with inflammation were curated from the published literature in our previous study.

#### Identification of phenotype-related PQRGs

The phenotype-related PQRGs could be obtained from the intersection of phenotype-specific DEGs and PQRGs.

#### Promoter analysis

Given the enrichment of PQS in promoter regions, which have the potential to influence gene expression, it is imperative to focus on the promoters of these phenotype-related PQRGs to assess the localization and quadruplex propensity of PQS.

#### Promoter sequence retrieval

The promoter sequences of the phenotype-related PQRGs can be retrieved from the Eukaryotic Promoter Database (EPD, http://epd.vital-it.ch). The promoter region could be defined as the genomic region spanning from −900 to +300 base pairs relative to the transcription start site.

#### PQS inference

The obtained promoter sequences can be run through web-based algorithms such as QGRS Mapper (https://bioinformatics.ramapo.edu/QGRS/index.php) and G4 Hunter (https://bioinformatics.ibp.cz/#/analyse/quadruplex) to analyze the presence of PQS within the promoter regions of the phenotype-related PQRGs. The QGRS Mapper could yield putative G4-forming G-rich sequences, along with their corresponding G-scores. Sequences with higher G-scores (typically above 30) are considered suitable candidates for further validation.

### Sequence-specific G4 assay for PQS

After acquiring the promoter PQS for these PQRGs, a pivotal step is to confirm their capability of G4 folding. Multiple assays could be utilized, including biochemical and biophysical approaches. In this section, we provide a concise introduction to these methods for the reference of researchers. The synthetic PQS of interest will be used to assess G4 formation with the following assays.

#### Biophysical method

1. Circular dichroism (CD): CD spectroscopy is a widely used method for investigating G-quadruplexes. It has the advantage of distinguishing between quadruplex topologies characterized by parallel and anti-parallel strand orientations. For a detailed protocol, please refer to the published work.
2. Analytical ultracentrifugation (AUC): AUC offers distinct advantages in the study of G4s. It provides measurements in solutions with well-defined and consistent compositions, making it highly sensitive to the absolute molecular weights and hydrodynamic shapes of molecules. AUC also minimizes the disruption of multi-stranded structures or ligand binding events, as the moving boundary of the G4 is not diluted or immobilized on a solid support. For detailed procedures, please refer to a published protocol.
3. Electrospray ionization mass spectrometry (ESI-MS): ESI-MS employs a “soft” ionization process, generating ions as an aerosol, which reduces the fragmentation of G4 structures. Importantly, the noncovalent interactions responsible for stabilizing G4s in solution are preserved in the gas phase. For detailed procedures, please refer to a published protocol.
4. X-ray crystallography: X-ray crystallography is a powerful technique for the direct visualization of G4 structures, including the metal ions at their core and other essential elements involved in G4 formation. Additionally, it allows for the visualization of ligand interactions with G4s. However, obtaining well-ordered crystals containing G4s can be challenging, which can limit the applicability of this method. For detailed procedures, please refer to a published protocol.
5. Nuclear magnetic resonance (NMR) spectroscopy: NMR spectroscopy has the unique advantage of identifying G4 structures under physiologically relevant ion conditions, characterized by the chemical shifts of amino peaks around 10.5–12 parts per million (ppm), distinct from other DNA conformations. Derivative methods include 19F NMR and in-cell NMR. 19F-labeled DNA is straightforward to prepare and lacks natural fluorine background signals, resulting in clear and simple 19F NMR spectra suitable for in-cell G4 identification. For detailed procedures, please refer to a published protocol.
6. Fluorescence resonance energy transfer (FRET) assay: FRET is a distance-dependent energy transfer process that provides insights into G4 structural dynamics at the molecular level. This technique enables the identification of G4 formation in target oligonucleotides under various ion or ligand treatment conditions, using a relatively simple experimental setup. For detailed procedures, please refer to a published protocol.
7. High-speed atomic force microscopy (HS-AFM): utilizing the DNA origami scaffold and HS-AFM system, researchers can directly visualize the formation and dissociation of G4s under various conditions at the single-molecule level. This technique offers valuable insights into the dynamics of G4 structures with high temporal resolution. For a comprehensive understanding of the experimental procedures, please refer to the published protocol.

#### Biochemical method

1. Electrophoretic mobility shift assay (EMSA) and dimethyl sulfate (DMS) footprinting: EMSA/DMS footprinting is a reliable method for the initial identification of G4 structures. It has a unique advantage in inferring the guanines involved in G-tetrad formation since their N7 position is inaccessible to methylation. These techniques are valuable not only for G4 structure identification but also for investigating G4-protein interactions. For detailed procedures, please refer to published protocols.
2. DNA polymerase stop assay: this assay is particularly suitable for identifying G4 formation in relatively long DNA segments (70–80 nucleotides). Additionally, it is valuable for the identification and evaluation of potential small molecules that interact with G4 structures. For comprehensive procedural details, please refer to the published protocols.
  For an in-depth review of the topic, we recommend referring to a published book.

### Determining the role of site-specific G4 folding in regulating gene expression

Once it has been confirmed with G4 assays that the PQS in the promoters of selected PQRGs can form G4 structures, the subsequent critical step is to determine how the formation of these G4 structures influences promoter activity. This empirical evidence is essential for substantiating the effect of G4 structures on the expression of PQRGs. To achieve this objective, a luciferase reporter system can be employed. Here is a concise outline of the procedure:

1. Promoter sequence mutation: begin by introducing mutations into the potential G4 structure-folding sequences within the promoter. This is achieved by substituting G/C base pairs with T/A base pairs to disrupt the structural basis of G4 folding.
2. Reporter plasmid construction: Reporter plasmids containing both the wild-type and mutated promoter sequences can be constructed, following established protocols as outlined in previous studies.
3. Transfection: subsequently, the constructed reporter plasmids can be transfected into the desired cells, following established standard protocols.
4. Treatment with G4 stabilizers: the cells transfected with the plasmids can be treated with G4 stabilizers to facilitate G4 stabilization.
5. Luciferase activity quantification: luciferase activity can be quantified to evaluate promoter function through a dual-luciferase assay system, using a multimode microplate reader.

#### Considerations for conducting experiments

To determine the specific mutant site in PQS for the reporter plasmid construction, a combination of computational inference and biophysical techniques can be employed to guide the decision-making process effectively. The *in vitro* biophysical techniques, such as FRET, can be instrumental in evaluating the impact of mutations on G4 formation.

As an example, in the “PQS Analysis in promoters of phenotype-specific genes” section, the high-scoring PQS “GGGTCACTTGGGAGAGGGCAGGG” is identified by the QGRS Mapper in the IL6 promoter, as depicted in Table 1.

**Table 1:** *In silico* characterization of PQS in the IL6 promoter using the QGRS Mapper

To confirm the role of this specific PQS in G4 formation, consider the following steps:

1. Mutation of PQS of interest: each guanine in the PQS sequence can be sequentially replaced with thymine. This results in a series of mutated sequences, such as “GTGTCACTTGGGAGAGGGCAGGG.”
2. *In silico* screen of mutant PQS with QGRS Mapper: the loss of G4-forming potential in the mutant sequence can be confirmed using the QGRS Mapper. If PQS is not identified or has a low propensity score in running QGRS Mapper, the subsequent biophysical assays should be applied to confirm this potentially destroyed mutation.
3. Biophysical verification with FRET: the potentially destructive mutation can be experimentally verified using a biophysical method, such as a FRET assay, to confirm the prevention of G4 structure formation.

### *In vitro* and *in vivo* validation of the role of G4 folding in phenotype regulation

Once the association between G4 structures and relevant phenotypes is established through algorithmic inference and bioinformatics analyses of sequencing data, the subsequent critical steps involve an experimental validation in both *in vitro* and *in vivo* settings. This validation aims to substantiate and elucidate the relationship between G4 structures and the observed phenotypes.

**Figure 3:**
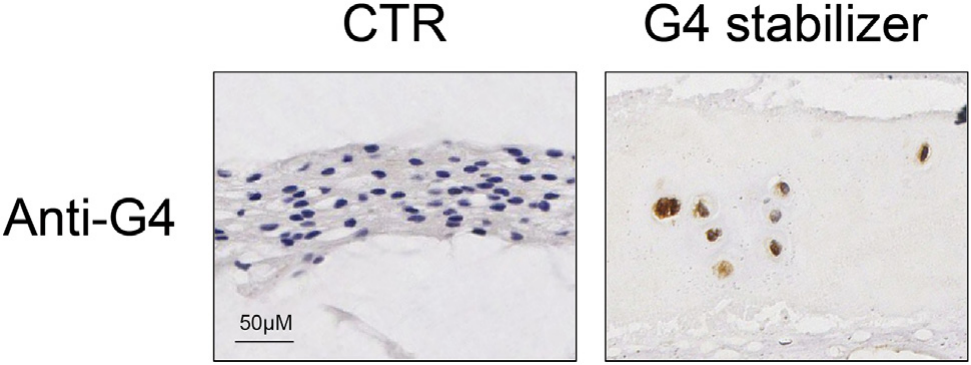
Representative immunohistochemistry staining for G4 The brown color represents anti-G4 immunostaining, indicating the presence of G4 structures.

1. Induction of specific phenotypes: the specific phenotypes could be induced with the relevant standard protocols in cell and animal models.
2. Characterizing genomic G4: the genomic G4 in response to the phenotype induction can be characterized using the optimal methods. For an experimental example, please refer to our previously published study or a published protocol. The optional methods include:
  a. Immunofluorescence analyses: genomic G4 formation can be visualized using G4-specific antibodies, such as BG4 or 1H6. This can be achieved by following the NP cell immunofluorescence protocol outlined in the previous section.
  b. Dot blot: the genomic G4 status can be quantitatively analyzed using G4 antibody BG4 after inducing a specific phenotype, following the procedure mentioned in the previous section.
  c. Immunohistochemistry (IHC): a specific phenotype or disease animal model can be constructed, and the necessary tissue can be collected. The effects of the phenotype on the genomic G4 state in the tissue can be quantitatively and positionally investigated using the G4-specific antibody BG4. Figure 3 visually shows how phenotype impacts genome-wide G4 folding in immunohistochemistry assays.

To investigate the influence of genomic G4 stabilization on specific phenotypes, appropriate G4 stabilizers can be applied in both cell and animal models. The selection of methods and testing markers may vary based on the research interest and the particular phenotype of interest. For example, in the context of inflammation research, methods such as western blot, quantitative real-time polymerase chain reaction (real-time qPCR), cell viability assay, enzyme-linked immunosorbent assay (ELISA), flow cytometry, and IHC can be employed to study the effect of genomic G4 folding on inflammation and apoptosis. The testing markers, such as IL-6 and TNF-α, can be examined to further elucidate the underlying mechanisms.

This guide offers fundamental principles and comprehensive instructions for conducting G4 research, aiding researchers in exploring G4 biology and disease development. By referring to this primer, researchers can effectively identify G4-regulated genes and phenotypes, phenotype-related G4, and specific G4 sequences in gene regulation. Thus, this primer may open valuable avenues for further investigation and advancing the understanding of G4 in regulation of biological process and disease development.

## Pitfalls and troubleshooting

### Regarding Greglist database

It’s crucial to emphasize that the sequences obtained from the Greglist database are PQS with the potential to form G4 structures. However, it’s critical to test the sequences with G4 assays, and the formation of G4 structures in cells is dynamic and influenced by various conditions.

### Regarding G4 stabilizers

When working with G4 stabilizers, it’s vital to notice the drug toxicity or other potential effects on molecular processes. Therefore, conducting concentration testing before their application becomes imperative, and the use of multiple G4 stabilizers can help mitigate potential confounding effects on downstream molecular processes.
